# Quota sampling method in online household surveys^☆^

**DOI:** 10.1016/j.mex.2022.101877

**Published:** 2022-10-08

**Authors:** Inas Nurfadia Futri, Tastaftiyan Risfandy, Mansor H. Ibrahim

**Affiliations:** a Faculty of Economics and Business, Center for Fintech and Banking, & GRISKA, Universitas Sebelas Maret, Surakarta, Indonesia; b INCEIF University, Kuala Lumpur, Malaysia

**Keywords:** Household surveys, Internet-based nonprobability sampling, Quota sampling, Enumerator

## Abstract

The Covid-19 pandemic has transformed people's activities in many aspects of human life, including how to conduct field surveys as part of research projects. The pandemic could substantially increase research expenses because the execution of surveys is limited by the activity restrictions imposed by the government. Field surveys during the pandemic are no longer flexible because for safety reasons, both the surveyor and the surveyed participant should be negative for Covid-19 by taking PCR tests. This condition could be problematic because research involving household samples is usually characterized by large sample sizes and wide coverage areas. The survey is also usually conducted directly in households to provide better and more accurate results. This article describes an alternative method for obtaining a representative sample of households using an internet-based survey at an affordable cost without ignoring the validity of the survey. This method can be widely implemented in social and economic field studies.

• The sampling method proposed for household research is quota sampling, with the strict application of proportions or controlled quotas.
• Recruiting local enumerators and selecting respondents based on the criteria is a tool to ease the validation process and quota control of respondents.

## Graphical abstract

**Figure:**
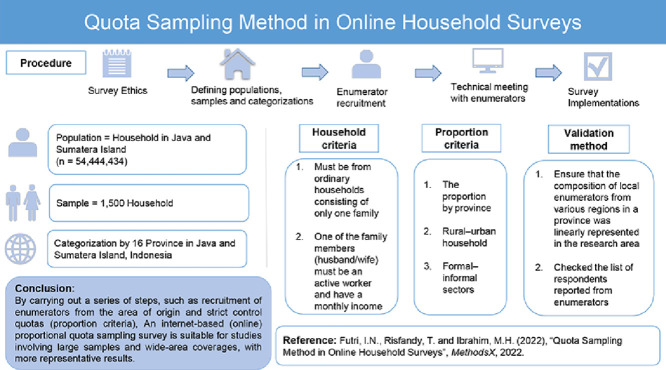

Specifications table

**Table:** 

**Subject area:**	Economics and Finance
**More specific subject area:**	Survey method
**Name of your method:**	Quota sampling Internet-based surveys
**Name and reference of original method:**	Moser, C. A.; Stuart, A. (1953). An Experimental Study of Quota Sampling, 116(4), 349–405. doi:10.2307/2343021
**Resource availability:**	N/A

## Method details

### Method details

Developing a new method in scientific research is useful because the correct method is needed to provide suitable recommendations to policymakers. The demand for a new approach to conducting scientific research increases when specific phenomena, such as the Covid-19 pandemic, occur. The limitations of all activities caused by the Covid-19 pandemic can undoubtedly be an obstacle to the research process, such as physical activity restrictions, long-distance travel, and other research activities involving crowds. All of these restrictions were imposed by the government to minimize the spread of Covid-19.

In research involving household subjects, researchers usually employ face-to-face (F2F) interviews or field surveys to obtain high-validity data [1]. These methods are also usually used by the government for national household sampling, such as when the BPS Statistics Indonesia surveyed consumption expenses in 2020 [2]. The limitations of activities during the pandemic can affect several research processes involving methods that require outdoor activities. The costs of carrying out a field survey will also increase because the surveyor and participant are required to implement strict rules imposed by the government, such as proof of negative Covid-19 tests. This condition is exacerbated when the research is to be carried out in different areas.

To minimize all risks and costs during the pandemic, researchers could alternatively use internet-based household surveys. Specifically, an online survey combined with the quota sampling method can be used to obtain data from households in different areas. Online survey tools, such as *Google Forms, SurveyMonkey,* and *TypeForm*, provide more flexibility than F2F interviews because the respondents can fill out the survey forms independently, anytime and anywhere. The use of internet-based questionnaires, when appropriately utilized, can be very effective [3].

Although online surveys can be very efficient, they require certain conditions to ensure data validity. This is because an online survey is usually open to everyone who wants to fill out the questionnaire, and people who do not meet the criteria can do so. In this paper, we provide an overview of an online survey design but with strict criteria to obtain valid respondents. Thus, a representative sampling method, such as probability sampling, is important for the credibility of the results. However, probability sampling in research is difficult and expensive, with large, scattered samples in some areas. Therefore, we propose quota sampling (a part of nonprobability sampling) instead of probability sampling [4].

Quota sampling was first introduced by Moser and Stuart in experimental research [5]. The quota sampling method is similar to stratified sampling, and it selects a sample from a population that has been divided into subgroups [6]. However, unlike stratified sampling, which relies on the random selection of each subgroup, quota sampling uses a convenience method within each subgroup. To increase the validity of the respondents, several specific criteria are applied in selecting samples after defining the population. This is known as the controlled quota.

The next section of this paper describes our process of conducting scientific research on households using internet-based surveys with quota sampling. This survey was carried out on households located on the islands of Java and Sumatra, the two islands with a population that dominates the Indonesian economy [7]. This procedure, of course, can also be carried out in other countries with relatively similar characteristics.

## Procedure

The procedure for conducting a survey consists of several stages:

1. Survey ethics
2. Defining populations, samples, and categorizations
3. Enumerator recruitment
4. Technical meeting with enumerators
5. Survey implementation

### Survey ethics

The first stage before starting the survey activity was to ensure that the process of conducting the survey and the survey instrument (questionnaire) did not violate the research ethics. We used Google Forms as the instrument. The ethics that were considered in the implementation of this survey included the transparency of the recruitment process that was carried out through social media, participant awareness of explicit privacy statements in online questionnaires, and protection of data confidentiality. These ethics were applied like other internet-based survey research described in other publications (see [8]).

### Defining populations, samples, and categorization

The population in this study was households domiciled in Java and Sumatra. The form of household based on the BPS Statistics Indonesia consists of ordinary and special households [9]. An ordinary household is a person or group of people who occupy part or whole physical buildings or censuses, and management of the same fulfills their daily needs. A household usually consists of a mother, father, and children. Conversely, a special household consists of a group of people from different families living in one physical building without their individual families. Examples of special households are people living in a dormitory, a correctional facility, or a group of people living in the same house (boarding house) with at least 10 occupants. We defined two criteria for our survey respondents. First, the respondents must be from ordinary households consisting of only one family. Second, one of the family members (husband/wife) must be an active worker and have a monthly income.

In using the quota sampling method, the population must first be divided into several groups. The categorization of the household population was divided by the provinces in which the households reside. In this case, the islands of Java and Sumatra have 16 provinces. To obtain sample results that were more representative of the population, we determined the proportion of the sample based on the percentage of total households in each province divided by the total number of households in Java and Sumatra, which was 54,444,434. The proportion results in each province were then multiplied by the target sample. In this study, we used data on the number of households from the BPS Statistics Indonesia to calculate the proportion of samples taken, and we defined the target sample of 1,500 households. Details of the number of samples taken by province are shown in Table 1.

**Table 1:** Samples taken by province.

No	Province	Total Household Population	Proportion: % of Total Population	Total sample: Proportion x 1,500	Final Sample (Rounded)
1	DI Aceh	1,231,058	0.023	33.917	34
2	North Sumatera	3,453,874	0.063	95.158	95
3	West Sumatera	1,291,397	0.024	35.579	36
4	Riau	1,522,700	0.028	41.952	42
5	Riau Island	521,100	0.010	14.357	15
6	Jambi	901,838	0.017	24.847	24
7	South Sumatera	2,052,499	0.038	56.548	57
8	Bangka Belitung Island	349,500	0.006	9.629	10
9	Bengkulu	509,000	0.009	14.023	14
10	Lampung	2,060,500	0.038	56.769	57
11	Banten	3,168,512	0.058	87.296	87
12	DKI Jakarta	2,758,709	0.051	76.005	76
13	West Java	13,231,615	0.243	364.545	364
14	Central Java	9,365,959	0.172	258.042	258
15	DI Yogyakarta	1,120,477	0.021	30.870	31
16	East Java	10,905,696	0.200	300.463	300
	Total	54,444,434			1500

#### Enumerator recruitment

**Fig. 1:**
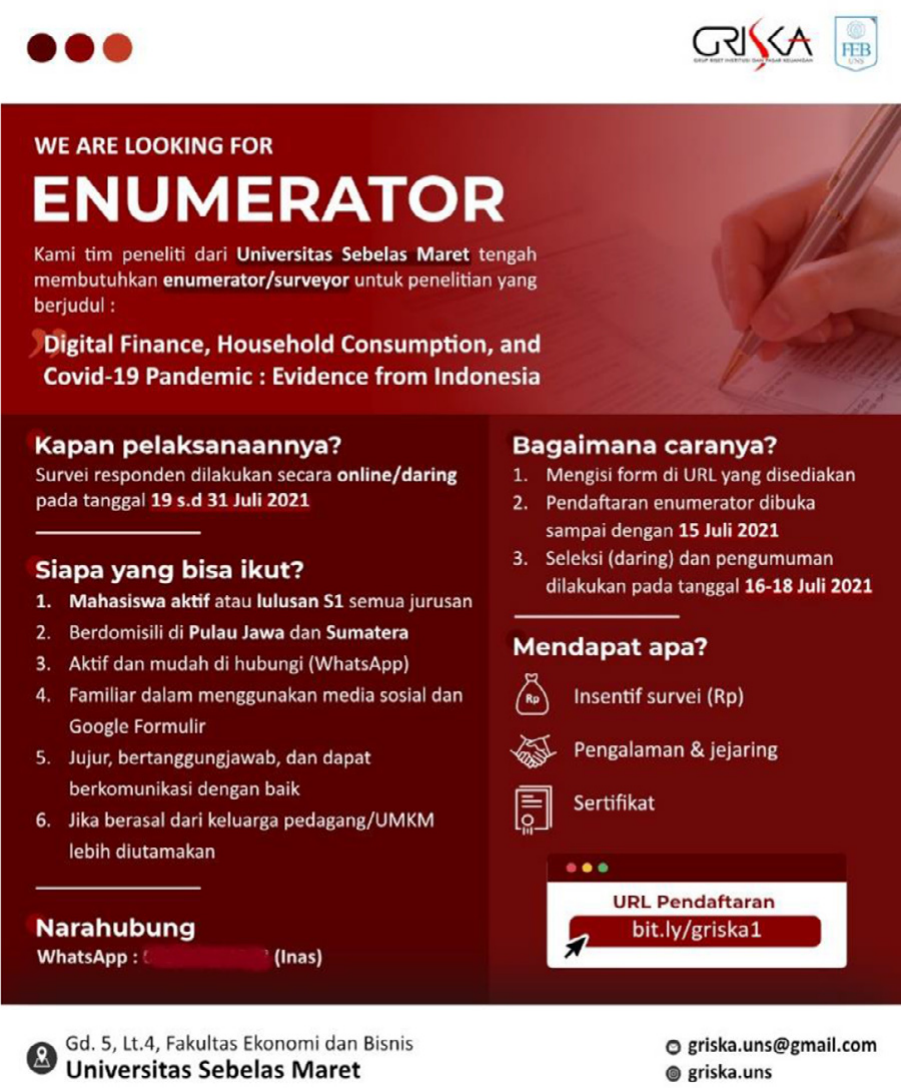
Flyer of enumerator recruitment.

Enumerators are individuals in charge of survey activities that help respondents answer questions and fill out questionnaires [10]. Even though the survey was conducted online, the recruitment of enumerators was essential to achieve the success of the target sample and ensure that the household respondents resided in the required area. At this stage, recruitment began by designing a recruitment announcement (in Bahasa Indonesia) distributed on open social media, such as Telegram, Instagram, Twitter, and some job portals. The enumerator recruitment was held on July 15–18, 2021, as shown in Fig. 1.

In looking for enumerator candidates, we defined the following criteria: (1) active or alumni of an undergraduate degree in any major, (2) domiciled in Java and Sumatra, (3) active and easy to contact, (4) familiar with social media and Google Forms, (5) reliable, responsible, and able to communicate, and (6) having a link with micro, small, and medium enterprises (MSME) was prioritized.

**Fig. 2:**
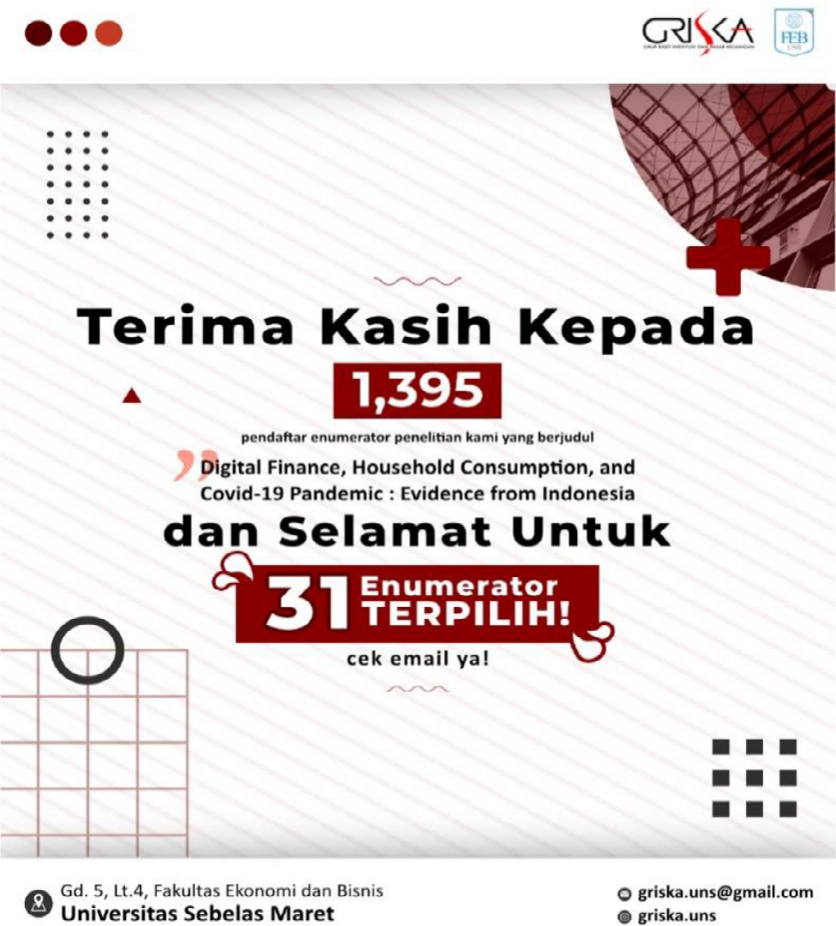
The announcement of selected enumerators.

Furthermore, we ensured that the selected enumerators were dispersed in 16 provinces to determine enumerators. In other words, an enumerator will distribute the questionnaire only to households in the same province where they reside. An enumerator should also distribute the questionnaire proportionally according to several criteria, such as the proportion of rural–urban, and formal–informal sectors. The standards we defined were essential for obtaining valid respondents and were relevant to our study. At the end of the recruitment process, we obtained 1,935 applicants and selected 31 enumerators from 16 provinces in Java and Sumatra (see Fig. 2). The announcement of acceptance was disseminated via email and on our official social media platform.

### Technical meeting with enumerators

The technical meeting was held online via the Zoom meeting platform. At this stage, there was a discussion regarding the details of the surveys, such as the objectives of the enumerators in this survey, respondent criteria, code and area distributions, and honorarium for the enumerators and respondents. As a rule, the respondents were not allowed to share the survey URL publicly. More specifically, they could share information about this survey publicly, but not the survey URL (Google form). This means that the enumerators had complete control over who their respondents were. In this way, the validity of the respondents can be maintained. The URL was distributed only to prospective respondents who met the criteria. In the end, the enumerator had full control to ensure that the proportion of respondents was in accordance with the proportion of rural-urban and formal-informal sector respondents.

In filling out the survey, the enumerators could help interview respondents, or the respondents could fill out the survey themselves (self-administered questionnaire). The enumerator had to instruct the respondents to enter the enumerator's assigned code. The code was required to make it easy to track the progress of the enumerator's work. Finally, the honorarium for each enumerator was wired to them at the end of the survey period after meeting the number of target respondents.

### Survey implementation

Online survey activities were carried out within two weeks, from July 19–31, 2021. In conducting this survey, five additional research assistants were involved; therefore, the total number of survey team members, including enumerators, was 36. The survey area distribution details are shown in Table 2.

**Table 2:** Distribution of the survey area.

No.	Province	Quota (Target)	Enumerators (Initials)	Enumerator Code
1	DI Aceh	34	RN	1RN
2	North Sumatra	48	ZIR	2ZIR
		47	SR	2SD
3	West Sumatra	36	WOF	3WOF
4	Riau	42	SR	4SR
5	Riau Island	15	AAA	5AAA
6	Jambi	24	SR	6RR
7	South Sumatra	57	AAB	7BS
8	Bangka Belitung Island	10	D	8BS
9	Bengkulu	14	ZY	9ZY
10	Lampung	57	MI	10MI
11	Banten	12	MIRH	11MIRH
		74	IM	11IM
12	DKI Jakarta	38	RM	12RM
		38	UA	12UM
13	West Java	45	SF	13SF
		13	LSS	13LSS
		45	SFA	13SFA
		45	F	13F
		45	NK	13NK
		77	IP	13IP
		45	AAPL	13AAPL
		45	CBW	13CBW
		5	INF	13INF
14	Central Java	40	AAF	14AAF
		40	AR	14AR
		40	SRM	14SRM
		40	HNL	14HNL
		40	ESR	14ESR
		40	BS	14BS
		18	INF	14INF
15	DI Yogyakarta	31	ES	15ES
16	East Java	43	DWP	16DWP
		43	DFSM	16DFSM
		43	UER	16UER
		43	SDS	16SDS
		43	YTA	16YTA
		43	DNS	16DNS
		42	ASR	16ARD
Total		**1,500**		

The enumerator was responsible for finding respondents with predetermined criteria according to the assigned quota for the number of respondents in each province or region. Then, the enumerator had to make a list of respondents and send it to the person in charge of the survey (a member of the research team). Making a list of incoming respondents serves to double-check the data from Google Forms. After verifying the data, an honorarium was sent to the respondents.

## Validation method

After the survey was completed, the next step was to validate the household sample to ensure that the sampled respondents met the required criteria. The first process relates to the selection of enumerator candidates. The quota sampling method is known to be at risk of geographic bias because of the tendency to select respondents to recruit people who live in their neighborhood [11]. To avoid this, enumerators were selected to ensure that the composition of the various regions in a province was linearly represented in the research area. In conducting the survey, some sections of the surveyed area were covered by more than one local enumerator.

Next, we checked the list of respondents reported on the Google sheet. The household respondent list report was a database in a Google sheet table stored in Google Drive and shared between the research assistants and the enumerators. The respondent list table for cross-checking contained the serial number, respondent's name, respondent's address (only village or city name), age, area information (rural/urban), occupation, and a description of respondent's relationships (neighbors, relatives, and others). Through the household respondent list report, enumerators and research assistants could check the composition of the respondents. It serves to ensure compliance with sample proportions, such as the ratio of villages to cities and the diversity of respondents’ occupations. The enumerator's work was deemed completed if the quota was completed according to the criteria. We did not check the respondent's identity card, as it is sensitive data for residents. However, we checked the duplication of the respondent's telephone number to ensure that the respondent did not double-fill or as a last measure proof of the validity of the respondent.

**Fig. 3:**
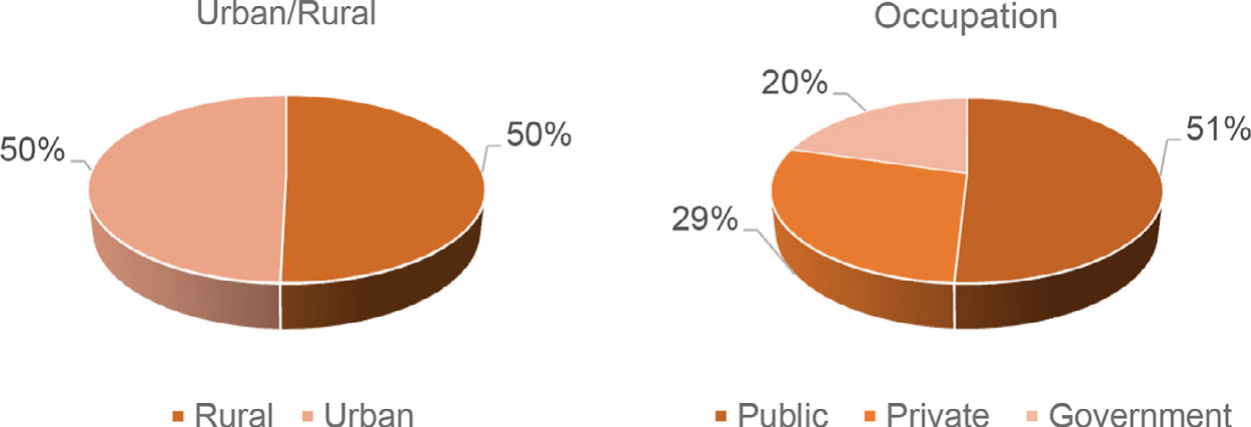
Proportion and characteristics of respondents.

The source of the bias in quota sampling was the non-representativeness of the sample. This is because the geographical diversity of the respondents was highly dependent on the enumerators. The enumerators usually selected respondents who were easily accessible ([4], [11]). However, we reduced bias in quota sampling by enriching geographic diversity and avoiding the concentration of respondents. The proportion of respondents in our study was based on rural and urban criteria (50:50), as can be seen in Fig. 3.

**Fig. 4:**
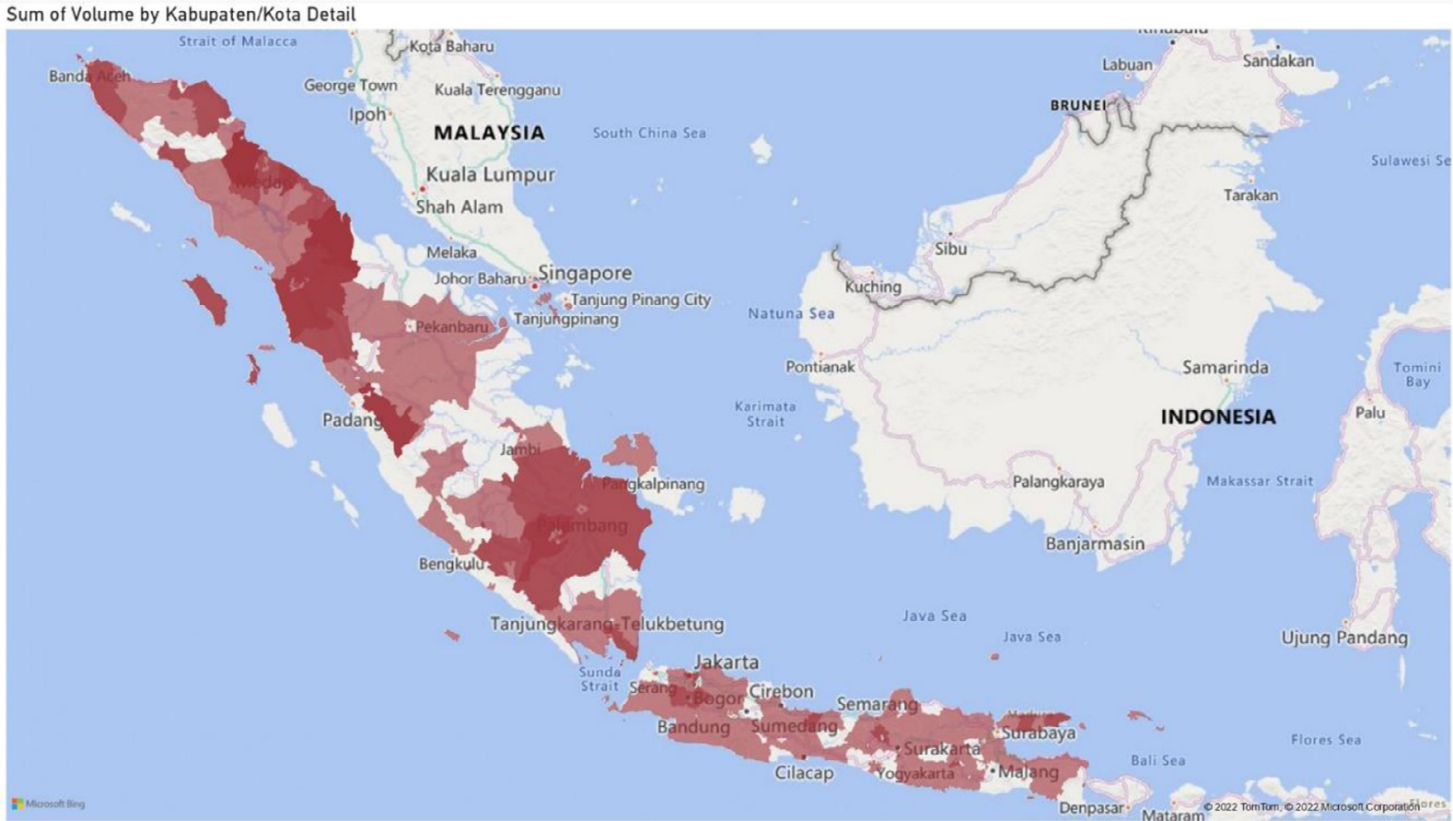
Respondent distribution location.

The bias of the quota sampling method is also minimized by distributing respondents based on regency/city locations on the islands of Java and Sumatera, as can be seen in Fig. 4. It can be seen that our sample is quite representative of the two provinces.

## Conclusion

Conducting a survey with a sample of households often requires a large amount of money, energy, and time to conduct F2F visits (field survey). The direct field survey was previously preferred in household research because it can ensure the validity of the household data and obtain a representative sample of the research area. An internet-based (online) proportional quota sampling survey was carried out as a combination of sampling methods with online instruments, reducing survey costs compared to F2F interviews. It is also more flexible than field surveys, especially during the Covid-19 pandemic, because people do not need to meet physically. By carrying out a series of steps, such as the recruitment of enumerators from the area of origin and strict controlled quotas (sample criteria/restrictions), this method is suitable for studies involving large samples and wide-area coverage, with more representative results. The success of this survey is certainly influenced by the excellent collaboration between the enumerators as implementers and the research team and the time and energy devoted to the process.

## Ethics statements

The participant aware of explicit privacy statements in online questionnaires and protection of data confidentiality. These ethics were applied like other internet-based survey research described in other publications. We also confirm that a) informed consent was obtained from participants or that participant data has been fully anonymized, and b) the platforms’ data redistribution policies were complied with the social media platform we use: Telegram, Twitter, and Instagram.

## Funding

This work is part of the research entitled “Digital finance, household consumption, and Covid-19 pandemic: Evidence from Indonesia,” funded by Research Grant Bank Indonesia 2021 with grant number 23/18/PKS/BINS/2021. The conclusions, opinions, and views expressed in this paper are the authors’ sole responsibility and do not necessarily reflect the official views of Bank Indonesia.

## CRediT authorship contribution statement

**Inas Nurfadia Futri:** Validation, Data curation, Software, Visualization, Writing – original draft. **Tastaftiyan Risfandy:** Conceptualization, Methodology, Investigation, Writing – review & editing. **Mansor H. Ibrahim:** Supervision, Validation.

## Declaration of conflict interests

The authors declare that they have no known competing financial interests or personal relationships that could have appeared to influence the work reported in this paper.

## Data Availability

Data will be made available on request.
